# Association of *SGK1* Polymorphisms With Susceptibility to Coronary Heart Disease in Chinese Han Patients With Comorbid Depression

**DOI:** 10.3389/fgene.2019.00921

**Published:** 2019-10-01

**Authors:** Wenxiu Han, Haixia Zhang, Xiaoxue Gong, Yujin Guo, Mengqi Yang, Hailiang Zhang, Xueyuan Zhou, Gongying Li, Yuanyuan Liu, Pei Jiang, Genquan Yan

**Affiliations:** ^1^Institute of Clinical Pharmacy & Pharmacology, Jining First People’s Hospital, Jining Medical University, Jining, China; ^2^Department of Cardiology, Jining First People’s Hospital, Jining Medical University, Jining, China; ^3^Department of Mental Health, Jining Medical University, Jining, China; ^4^Research Center of Basic Medical Sciences, Tianjin Medical University, Tianjin, China; ^5^Department of Pharmacy, Shandong Provincial Hospital affiliated to Shandong University, Jinan, China

**Keywords:** serum/glucocorticoid-regulated kinase 1, coronary heart disease, depression, polymorphism, stress

## Abstract

There is a strong link between heart disease and depression, both of which are closely related to lifetime stress exposure. Serum/glucocorticoid-regulated kinase 1 (*SGK1*) is a stress-responsive gene with a pivotal role in both the heart and brain. To determine the role of *SGK1* polymorphisms (rs2758151, rs1743963, rs9493857, rs1763509, rs9376026, and rs9389154) in susceptibility to comorbid coronary heart disease (CHD) and depression, we conducted a hospital-based case–control study involving 257 CHD cases (including 69 cases with depression and 188 cases without depression) and 107 controls in a Chinese Han population. Six single-nucleotide polymorphisms (SNPs) in the *SGK1* gene were successfully genotyped by polymerase chain reaction–ligase detection reaction (PCR-LDR) assay. Our results showed no significant differences in *SGK1* genetic polymorphisms between CHD patients and controls, whereas significant associations were observed between *SGK1* SNPs (rs1743963 and rs1763509) and the development of depression in CHD patients (*P* = 0.018 by genotype, *P* = 0.032 by allele; *P* = 0.017 by genotype, *P* = 0.003 by allele, respectively). However, none of these associations remained significant after Bonferroni correction (*P* = 0.054 for rs1743963; *P* = 0.051 for rs1763509). Interestingly, both the GG genotype of *SGK1* rs1743963 and AA genotype of *SGK1* rs1763509 were associated with a higher risk of depression in CHD patients; for rs1763509, the Patient Health Questionnaire-9 (PHQ-9) scores in the carriers of the risk genotype for comorbid depression, AA, were significantly higher than in GG and AG carriers (*P* = 0.008). Notably, haplotype analysis indicated that haplotype GGA significantly increased the risk of depression in CHD patients (*P* = 0.011, odds ratio (OR) = 1.717, 95% confidence interval (CI) = 1.132–2.605), whereas haplotype AAG may be a protective factor for CHD patients with comorbid depression (*P* = 0.038, OR = 0.546, 95% CI = 0.307–0.972). It should be noted that only the significance of haplotype GGA survived after Bonferroni adjustment (*P* = 0.044) and that no significant differences were found for other *SGK1* SNPs (rs2758151, rs9493857, rs9376026, and rs9389154) between CHD patients with and without depression. These findings, for the first time, elucidate the important role of *SGK1* variants in the comorbidity of CHD and depression.

## Introduction

Coronary heart disease (CHD) is among the most common chronic diseases, with a severe impact on human health and quality of life. It is also considered to be a psychosomatic disease. The poor prognosis and high sudden death rate associated with CHD may also result in the development of comorbid psychological complications such as anxiety and depression. Accumulating evidence shows that CHD patients suffer from depression to some extent and that the prevalence of depression in CHD patients is twice as high as in the general population (Follath, 2003). As a result, CHD with comorbid depression has become a concern worldwide. An increasing number of studies show that CHD and depression share common risk mechanisms, including inflammation (Shimohina et al., 2015), autonomic dysfunction (Drago et al., 2007), hypothalamus–pituitary–adrenocortical axis dysfunction (Lederbogen and Strohle, 2012), and enhancement of platelet aggregation activity (Tseng et al., 2010). Multiple genetic factors have also become the new focus of scientific studies. Emerging data suggest that genetic defects in 5-hydroxytryptamine (5-HT) (Golimbet et al., 2012), apolipoprotein E (ApoE) (Fritze et al., 2011), endothelial NOS (eNOS) (Salimi et al., 2012; Talarowska et al., 2012), and plasminogen-activator inhibitor-1 (PAI-1) (Lahlou-Laforet et al., 2006) may be related to the risk of CHD with comorbid depression.

A member of the serum/glucocorticoid-regulated kinase (SGK) family, serum/glucocorticoid-regulated kinase 1 (*SGK1*) regulates several ion channels and participates in many cellular reactions, including cell growth, proliferation, migration, survival, and apoptosis (Talarico et al., 2016). A recent study showed that *SGK1* contributes to the regulation of renal Na^+^ reabsorption, K^+^ secretion, and blood pressure (Valinsky et al., 2018). Given the close association between high blood pressure levels and risk of CHD, it is reasonable to speculate that *SGK1* is related to the risk of CHD. Additionally, *SGK1* plays a vital part in the regulation of neuronal activity, proliferation, and apoptosis and thus is a key determinant of susceptibility to mental illness. As the downstream target molecule of the glucocorticoid receptor (GR), *SGK1* is involved in the development of depression *via* the glucocorticoid signaling pathway. There is also growing evidence indicating that *SGK1* stimulates the production of pro-inflammatory cytokines and oxidants (Lang et al., 2010), which are also closely related to depression. Taking into consideration the complex relationships among *SGK1*, CHD, and depression, we hypothesize that *SGK1* may be a co-pathogenic gene underlying the comorbid mechanisms of CHD and depression. Thus, to further evaluate the role of *SGK1* single-nucleotide polymorphisms (SNPs) in susceptibility to CHD with comorbid depression, we carried out a case–control study involving 257 CHD patients with or without depression and 107 controls.

## Materials and Methods

### Subjects

A total of 257 CHD patients were recruited at the outpatient clinic of the Jining First People’s Hospital in Shandong Province, China. For all patients, the diagnosis of CHD was made by at least two experienced cardiologists and confirmed using coronary angiography results (significant coronary artery stenosis ≥ 50% in at least one of the three major coronary arteries or major branches). Those with valvular heart disease, severe autoimmunity disease, cancer, or severe liver and/or kidney disease were excluded. In addition, all CHD patients with or without depression were assessed by at least two experienced psychiatrists according to DSM-5 (5th edition of the *Diagnostic and Statistical Manual of Mental Disorders*) criteria for major depressive disorder, which is characterized by significant depressed mood and anhedonia. Then, the severity of depressive symptoms was scored by Patient Health Questionnaire-9 (PHQ-9), a nine-item questionnaire that is commonly used in outpatients. The scale uses a cutoff score for depression analysis of greater than or equal to 5 (Duko et al., 2018). The 107 age- and sex-matched healthy controls were adults without CHD who had undergone a series of assessments including clinical physical examination, radiographic chest examination, electrocardiogram, and evaluation of medical history. Our study received approval from the medical ethics committee of the Jining First People’s Hospital, and informed consents were obtained from all participants.

### Genetic Studies (DNA Isolation and Genotyping)

Genomic DNA was isolated from whole blood using a TIANamp Blood DNA Kit (TIANGEN, China) following the manufacturer’s instructions. The genotypes of polymorphisms were identified by polymerase chain reaction–ligase detection reaction (PCR-LDR) assay. All primer sequences for both PCR and LDR are shown in **Table 1**. After identification using 1.5% agarose gel and a multiplex ligase detection reaction with an LDR probe, products were determined by direct sequencing with a DNA sequencer. To ensure the quality of genotyping, random DNA samples accounting for not less than 10% of the total subjects were genotyped twice. Genotyping quality assessment of the SNPs tested is presented in **Supplementary Table 1**.

**Table 1:** Primers of target gene used in the PCR.

SNP	Ancestor allele	Primer sequence	Product size (bp)
rs2758151	C	F: 5′-ACGTTGGATGGGTAAAGGG​AACTTCAGACG-3′	108
		R: 5′-ACGTTGGATGGAAGAATCTT​AGAGCTTCC-3′	
rs1743963	A	F: 5′-ACGTTGGATGAGCCAGTGCT​GGCCGGGAA-3′	88
		R: 5′-ACGTTGGATGGTGGTAACTT​GTAACTGCCC-3′	
rs9493857	A	F: 5′-ACGTTGGATGGATTATTGTTG​CAATGGAAGG-3′	100
		R: 5′-ACGTTGGATGGTGATCATTTG​ATTACTGC-3′	
rs1763509	G	F: 5′-ACGTTGGATGGGAGTAGAGA​GATGAGTTTC-3′	120
		R: 5′-ACGTTGGATGTTACACTGAAA​GAAGTATG-3′	
rs9376026	C	F: 5′-ACGTTGGATGCTCAGTACTCTT​AATGGATG-3′	95
		R: 5′-ACGTTGGATGCACCTATTAGAT​GTGTGGTC-3′	
rs9389154	G	F: 5′-ACGTTGGATGGACCACTTACT​AAAAGGAAGC-3′	120
		R: 5′-ACGTTGGATGTCAGGCTTCCTT​GAGTTTGG-3′	

PCR, polymerase chain reaction; SNP, single-nucleotide polymorphism.

### Statistical Analysis

Demographic and clinical characteristics of the study population were evaluated by *t*-test (for continuous variables) and Pearson’s *χ* ^2^-test (for categorical variables). Genotype distributions and allele frequencies of CHD patients and controls were analyzed by Pearson’s *χ* ^2^-test. To evaluate the quality of the genotyping data, the SHEsis online haplotype analysis software (http://analysis.bio-x.cn/myAnalysis.php) was applied to calculate the linkage disequilibrium and check Hardy–Weinberg equilibrium in controls based on Pearson’s *χ* ^2^-test. Additionally, the SHEsis online haplotype analysis software was also performed for calculating the probability of obtaining a difference in the haplotype frequencies observed between patients and controls and for analyzing the haplotype frequencies and probabilities. Bonferroni adjustment was applied to correct for multiple comparisons. Odds ratios (ORs) and 95% confidence intervals (95% CIs) were also calculated. Differences in PHQ-9 scores among different genotypic individuals were assessed using one-way analysis of variance (ANOVA) or Student’s *t*-test, when appropriate. All analyses were carried out using SPSS (version 17.0), and *P* < 0.05 was defined as statistically significant.

## Results

### Basic Characteristics of Study Participants

The demographic and clinical characteristics of the study participants are described in **Table 2**. There were no significant differences between the CHD and control groups in terms of age, gender, body mass index (BMI), and smoking or drinking (*P* > 0.05). Then, CHD patients were further divided into CHD+D and CHD-D groups according to whether comorbid depression was present. There were still no significant differences concerning the basic characteristics between groups (*P* > 0.05).

**Table 2:** Demographic and clinical characteristics of the study participants.

Variables	CHD (*n* = 257)	Controls (*n* = 107)	*P*-value	CHD+D (*n* = 69)	*P*-value	CHD-D (*n* = 188)	*P*-value
Age (years)	51.04 ± 6.854	49.84 ± 7.965	0.148^a^	51.26 ± 6.795	0.224^b^	51.24 ± 6.897	0.982^c^
Gender (M/F, *n*)	136/121	49/58	0.216^a^	32/37	0.940^b^	104/84	0.203^c^
Smoking (*n*, %)	89 (34.6)	32 (29.9)	0.383^a^	21 (30.4)	0.941^b^	68 (36.2)	0.392^c^
Drinking (*n*, %)	99 (38.5)	30 (28.0)	0.057^a^	22 (31.9)	0.585^b^	77 (41.0)	0.185^c^
BMI (kg/m^2^)	23.73 ± 2.821	23.37 ± 2.332	0.245^a^	23.68 ± 2.543	0.403^b^	24.17 ± 2.938	0.218^c^

^a^CHD versus controls, ^b^CHD+D versus controls, ^c^CHD+D versus CHD-D. BMI, body mass index; CHD, coronary heart disease; CHD+D, CHD with depression; CHD-D: CHD without depression.

### Hardy–Weinberg Equilibrium Analysis

The locations of the *SGK1* gene and six SNPs are presented in **Supplementary Table 2**. The genotypes of all *SGK1* SNPs in control groups were in Hardy–Weinberg equilibrium based on the *χ* ^2^-test results (rs2758151: *χ* ^2^ = 0.020, *P* = 0.887; rs1743963: *χ* ^2^ = 0.115, *P* = 0.734; rs9493857: *χ* ^2^ = 0.472, *P* = 0.492; rs1763509: *χ* ^2^ = 0.080, *P* = 0.778; rs9376026: *χ* ^2^ = 2.909, *P* = 0.088; rs9389154: *χ* ^2^ = 0.072, *P* = 0.789), suggesting that the groups are representative of the population.

### *SGK1* Polymorphisms

Frequency distributions of genotypes and alleles of six SNPs in CHD patients and controls are shown in **Tables 3** and **4**. Our results suggest the absence of a significant relationship between *SGK1* SNPs and CHD risk. However, significant differences were found between CHD patients with and without depression in the genotype distribution and allele frequency of rs1743963 (A > G) and rs1763509 (G > A). For rs1743963, CHD patients with the GG genotype showed a significant susceptibility to depression (*χ* ^2^ = 7.988, *P* = 0.018). Thus, the G allele may be a risk factor in the development of depression in CHD patients (*χ* ^2^ = 4.572, *P* = 0.032). For rs1763509, the AA genotype and A allele were associated with a higher risk of depression in CHD patients (*χ* ^2^ = 8.118, *P* = 0.017 by genotype; *χ* ^2^ = 8.974, *P* = 0.003 by allele). However, the significance of the genotype distribution frequency could not be confirmed after strict Bonferroni adjustment (*P* = 0.054 for rs1743963; *P* = 0.051 for rs1763509). Interestingly, when subdividing these samples into GG and AA + AG groups, statistical analysis showed that carriers with allele A were more likely to have comorbid depression (*χ* ^2^ = 4.238, *P* = 0.04, OR = 4.213, 95% CI = 0.961–18.466). However, the other four SNPs, rs2758151, rs9493857, rs9376026, and rs9389154, were not significantly related to the risk of depression in CHD patients (as shown in **Tables 5** and **6**).

**Table 3:** Genotype distribution of *SGK1* gene polymorphisms in CHD and control group.

SNP	Genotype	CHD (*n* = 257, %)	Controls (*n* = 107, %)	OR (95% CI)	*χ* ^2^	*P*-value
rs2758151	CC	75 (29.2)	30 (28.0)			
(C > T)	CT	131 (51.0)	54 (50.5)			
	TT	51 (19.8)	23 (21.5)		0.139	0.933
	CT + TT	182 (70.8)	77 (72.0)	1.058 (0.641–1.744)	0.048	0.826
rs1743963	AA	41 (15.9)	11 (10.3)			
(A > G)	AG	121 (47.1)	49 (45.8)			
	GG	95 (37.0)	47 (43.9)		2.667	0.264
	AG + GG	216 (84.0)	96 (89.7)	1.657 (0.816–3.361)	1.986	0.159
rs9493857	AA	14 (5.4)	3 (2.8)			
(A > G)	AG	85 (33.1)	36 (33.6)			
	GG	158 (61.5)	68 (63.6)		1.308	0.520
	AG + GG	243 (94.6)	104 (97.2)	1.997 (0.562–7.097)	0.666	0.414
rs1763509	GG	23 (9.0)	5 (4.7)			
(G > A)	AG	89 (34.6)	34 (31.8)			
	AA	145 (56.4)	68 (63.5)		2.197	0.333
	AG + AA	234 (91.0)	102 (95.3)	2.005 (0.742–5.422)	1.946	0.163
rs9376026	CC	176 (68.5)	66 (61.7)			
(C > T)	CT	74 (28.8)	32 (29.9)			
	TT	7 (2.7)	9 (8.4)		5.546	0.062
	CT + TT	81 (31.5)	41 (38.3)	1.350 (0.843–2.160)	1.568	0.211
rs9389154	GG	53 (20.6)	30 (28.0)			
(G > A)	AG	124 (48.3)	52 (48.6)			
	AA	80 (31.1)	25 (23.4)		3.402	0.182
	AG + AA	204 (79.4)	77 (72.0)	0.667 (0.397–1.120)	2.360	0.125

CHD, coronary heart disease; CI, confidence interval; OR, odds ratio; SNP, single-nucleotide polymorphism.

**Table 4:** Allele distribution of *SGK1* gene polymorphisms in CHD and control group.

SNP	Allele	CHD (2*n* = 514, %)	Controls (2*n* = 214, %)	OR (95% CI)	*χ* ^2^	*P*-value
rs2758151	C	281 (54.7)	114 (53.3)			
(C > T)	T	233 (45.3)	100 (46.7)	1.058 (0.768–1.457)	0.119	0.730
rs1743963	A	203 (39.5)	71 (33.2)			
(A > G)	G	311 (60.5)	143 (66.8)	1.315 (0.940–1.838)	2.568	0.109
rs9493857	A	113 (22.0)	42 (19.6)			
(A > G)	G	401 (78.0)	172 (80.4)	1.154 (0.776–1.716)	0.501	0.479
rs1763509	G	135 (26.3)	44 (20.6)			
(G > A)	A	379 (73.7)	170 (79.4)	1.376 (0.936–2.023)	2.651	0.103
rs9376026	C	426 (82.9)	164 (76.6)			
(C > T)	T	88 (17.1)	50 (23.4)	1.476 (0.998–2.182)	3.834	0.05
rs9389154	G	230 (44.7)	112 (52.3)			
(G > A)	A	284 (55.3)	102 (47.7)	0.738 (0.536–1.015)	3.494	0.062

CHD, coronary heart disease; CI, confidence interval; OR, odds ratio; SNP, single-nucleotide polymorphism.

**Table 5:** Genotype distribution of *SGK1* gene polymorphisms in CHD+D and CHD-D group.

SNP	Genotype	CHD+D (*n* = 69, %)	CHD-D (*n* = 188, %)	OR (95% CI)	*χ* ^2^	*P*-value^a^	*P*-value^b^
rs2758151	CC	21 (30.4)	54 (28.7)				
(C > T)	CT	33 (47.8)	99 (52.7)				
	TT	15 (21.8)	35 (18.6)		0.533	0.766	
	CT + TT	48 (69.6)	134 (71.3)	1.086 (0.594–1.983)	0.072	0.789	
rs1743963	AA	10 (14.5)	31 (16.5)				
(A > G)	AG	24 (34.8)	97 (51.6)				
	GG	35 (50.7)	60 (31.9)		7.988	**0.018**	0.054
	AG + GG	59 (85.5)	157 (83.5)	1.165 (0.538–2.524)	0.150	0.698	
rs9493857	AA	3 (4.3)	12 (6.4)				
(A > G)	AG	18 (26.1)	67 (35.6)				
	GG	48 (69.6)	109 (58.0)		2.924	0.232	
	AG + GG	66 (95.7)	176 (93.6)	0.667 (0.182–2.437)	0.100	0.752	
rs1763509	GG	2 (2.9)	21 (11.2)				
(G > A)	AG	19 (27.5)	70 (37.2)				
	AA	48 (69.6)	97 (51.6)		8.118	**0.017**	0.051
	AG + AA	67 (97.1)	167 (88.8)	4.213 (0.961–18.466)	4.238	**0.04**	
rs9376026	CC	48 (69.6)	131 (69.7)				
(C > T)	CT	20 (29.0)	51 (27.1)				
	TT	1 (1.4)	6 (3.2)		0.702	0.704	
	CT + TT	21 (30.4)	57 (30.3)	0.995 (0.546–1.812)	0.000	0.986	
rs9389154	GG	10 (14.5)	43 (22.9)				
(G > A)	AG	38 (55.1)	87 (46.3)				
	AA	21 (30.4)	58 (30.8)		2.524	0.283	
	AG + AA	59 (85.5)	145 (77.1)	0.572 (0.270–1.212)	2.165	0.141	

^a^P-value without adjustment; ^b^P-value after Bonferroni adjustment for multiple comparisons; P-value < 0.05 has been bolded. CHD, coronary heart disease; CI, confidence interval; OR, odds ratio; SNP, single-nucleotide polymorphism.

**Table 6:** Allele distribution of *SGK1* gene polymorphisms in CHD+D and CHD-D group.

SNP	Allele	CHD+D (2*n* = 138, %)	CHD-D (2*n* = 376, %)	OR (95% CI)	*χ* ^2^	*P*-value
rs2758151	C	75 (54.3)	207 (55.1)			
(C > T)	T	63 (45.7)	169 (44.9)	0.972 (0.657–1.438)	0.020	0.887
rs1743963	A	44 (31.9)	159 (42.3)			
(A > G)	G	94 (68.1)	217 (57.7)	1.565 (1.036–2.364)	4.572	**0.032**
rs9493857	A	24 (17.4)	91 (24.2)			
(A > G)	G	114 (82.6)	285 (75.8)	0.659 (0.400–1.086)	2.696	0.101
rs1763509	G	23 (16.7)	112 (29.8)			
(G > A)	A	115 (83.3)	264 (70.2)	2.121 (1.288–3.495)	8.974	**0.003**
rs9376026	C	116 (84.1)	313 (83.2)			
(C > T)	T	22 (15.9)	63 (16.8)	1.061 (0.625–1.803)	0.048	0.826
rs9389154	G	58 (42.0)	173 (46.0)			
(G > A)	A	80 (58.0)	203 (54.0)	0.851 (0.574–1.262)	0.647	0.421

P-value < 0.05 has been bolded. CHD, coronary heart disease; CI, confidence interval; SNP, single-nucleotide polymorphism.

### Association of *SGK1* Polymorphisms With Severity of Depressive Symptoms

**Figure 1:**
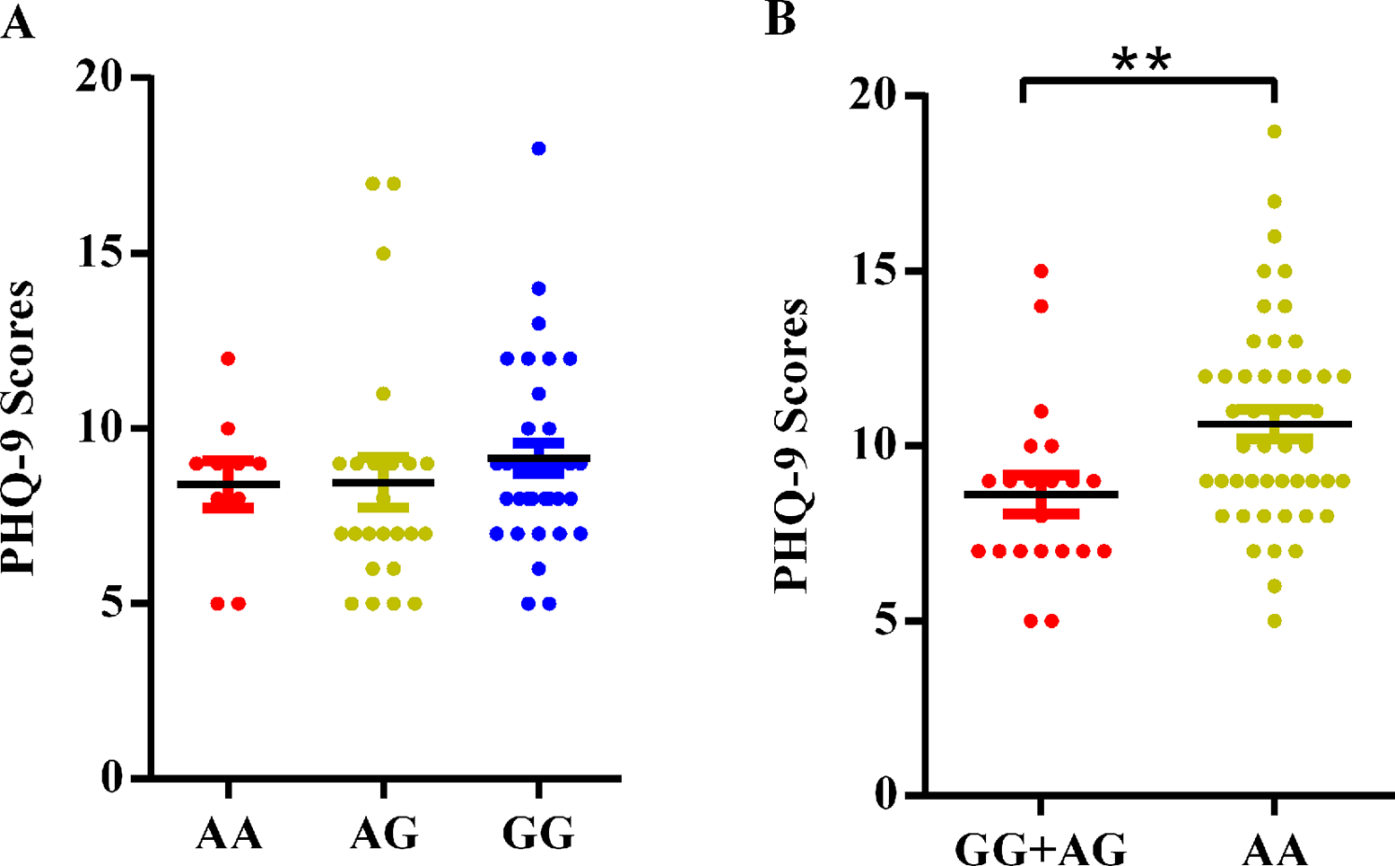
Association of *SGK1* polymorphisms and PHQ-9 scores in CHD patients with comorbid depression. **(A)** rs1743963 and **(B)** rs1763509. **P < 0.01. CHD, coronary heart disease; PHQ-9, Patient Health Questionnaire-9.

As shown in **Figure 1A**, no significant differences in PHQ-9 scores were observed among the three genotypes of rs1743963 (*P* > 0.05). For rs1763509 (**Figure 1B**), GG and AG carriers were combined because of the few GG carriers. The PHQ-9 scores in the AA carriers, the risk genotype for comorbid depression, were significantly higher than GG and AG carriers (10.63 ± 2.900 versus 8.62 ± 2.500, *P* = 0.008).

### Haplotype Analysis

**Figure 2:**
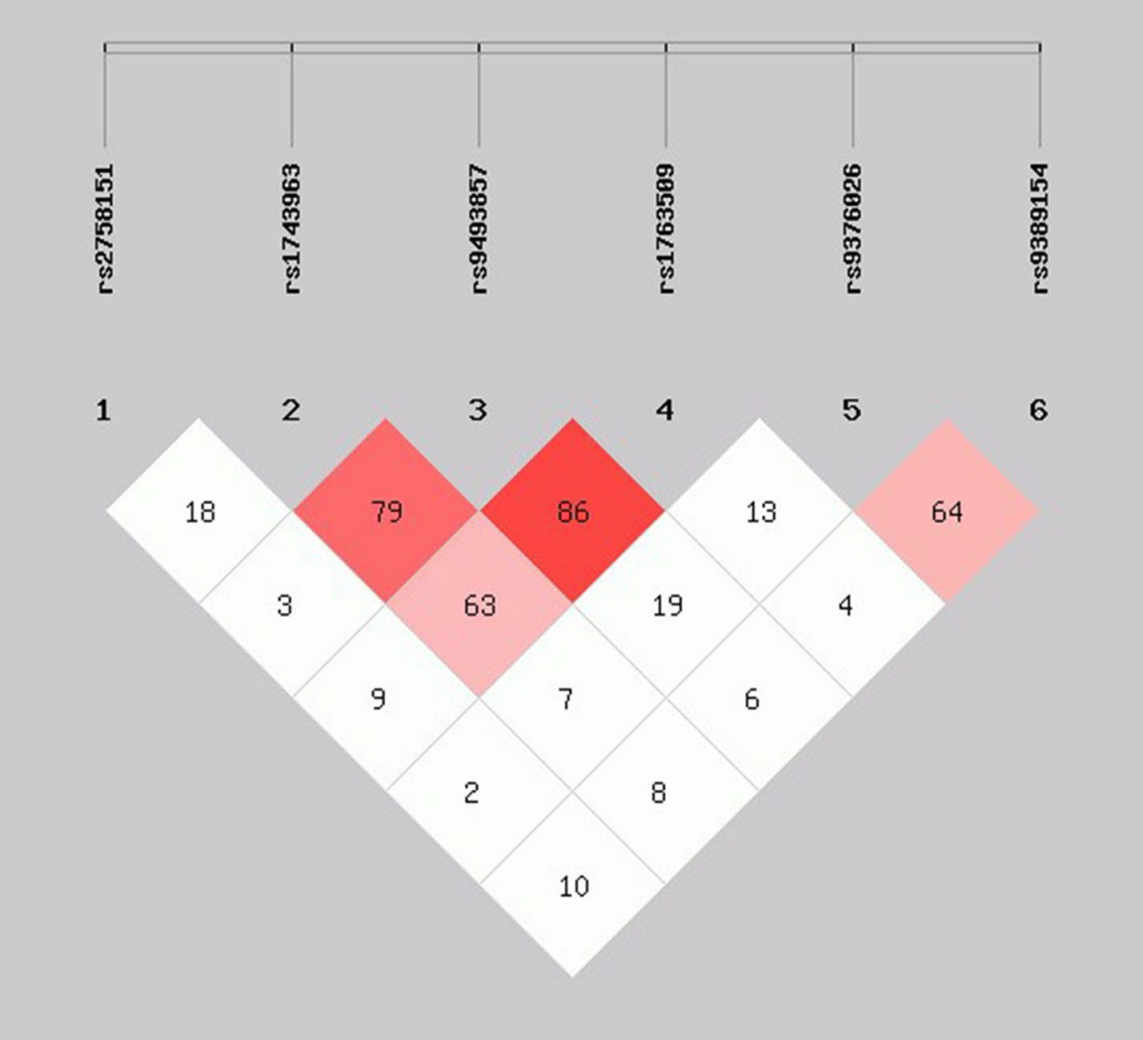
Linkage disequilibrium pattern between three SNPs, rs1743963, rs9493857, and rs1763509, in CHD patients and healthy controls. CHD, coronary heart disease; SNP, single-nucleotide polymorphism.

As shown in **Figure 2**, the LD block in the *SGK1* gene on chromosome 6 comprised rs1743963, rs9493857, and rs1763509, with a strong linkage (rs1743963/rs9493857: *D*′ = 0.793, *r* ^2^ = 0.282; rs9493857/rs1763509: *D*′ = 0.869, *r* ^2^ = 0.675; rs1743963/rs1763509: *D*′ = 0.633, *r* ^2^ = 0.201). Haplotype frequencies indicated that there were no significant differences of haplotype distribution between CHD patients and healthy controls (as shown in **Table 7**). Interestingly, haplotype analysis of the CHD+D and CHD-D groups revealed that haplotype GGA significantly increased the risk of depression in CHD patients (*P* = 0.011, OR = 1.717, 95% CI = 1.132–2.605), while haplotype AAG may be a protective factor against comorbid depression in CHD patients (*P* = 0.038, OR = 0.546, 95% CI = 0.307–0.972). After Bonferroni adjustment, only the haplotype GGA remained significantly associated with the susceptibility to depression in CHD patients (*P* = 0.044) (**Table 8**).

**Table 7:** Haplotype frequencies for *SGK1* polymorphisms in CHD and control group.

Haplotype (rs1743963/rs9493857/rs1763509)	CHD 2*n* = 514 (%)	Controls 2*n* = 214 (%)	OR (95% CI)	*P*-value
AAG	91.39 (17.8)	32.78 (15.3)	1.219 (0.787–1.888)	0.374
AGA	95.93 (18.7)	35.10 (16.4)	1.192 (0.778–1.827)	0.418
GGA	280.53 (54.6)	131.82 (61.6)	0.760 (0.542–1.066)	0.111
GGG	16.88 (3.3)	5.05 (2.4)	1.428 (0.521–3.913)	0.487

CHD, coronary heart disease; CI, confidence interval; OR, odds ratio. Haplotypes were omitted if the estimated haplotype frequency was <3%.

**Table 8:** Haplotype frequencies for *SGK1* polymorphisms in CHD+D and CHD-D group.

Haplotype (rs1743963/rs9493857/rs1763509)	CHD+D 2*n* = 138 (%)	CHD-D 2*n* = 376 (%)	OR (95% CI)	*P*-value^a^	*P*-value^b^
AAG	16.53 (12.0)	73.74 (19.6)	0.546 (0.307–0.972)	**0.038**	0.152
AGA	24.57 (17.8)	72.42 (19.3)	0.894 (0.537–1.487)	0.665	
GGA	85.30 (61.8)	184.04 (48.9)	1.717 (1.132–2.605)	**0.011**	**0.044**
GGG	4.12 (3.0)	21.35 (5.7)	0.505 (0.172–1.477)	0.204	

CHD, coronary heart disease; CI, confidence interval; OR, odds ratio. Haplotypes were omitted if the estimated haplotype frequency was <3%. ^a^P-value without adjustment; ^b^P-value after Bonferroni adjustment for multiple comparisons; P-value < 0.05 has been bolded.

## Discussion

The gene encoding human *SGK1* is located in chromosome 6q23.2. *SGK1* transcripts have been found in virtually all tissues tested (Raikwar et al., 2008). A key regulatory enzyme, *SGK1* was originally described as being involved in the hormonal regulation of several ion channels (Lang et al., 2011; Chraibi and Renauld, 2014). *SGK1* is linked to the regulation of Na^+^ and K^+^ transport in epithelial cells (Valinsky et al., 2018). Studies have shown that dysregulation of *SGK1* causes renal Na^+^ retention and enhancement of cardiac output, followed by elevated blood pressure (Kawarazaki et al., 2012; Nakano et al., 2013). Several *SGK1* gene variants have been shown to affect blood pressure (Busjahn et al., 2002; Rao et al., 2013). Accumulating strong evidence indicates a direct connection between *SGK1* and cardiovascular development *via* involvement in the phosphatidylinositol 3-kinase (Catela et al., 2010) and ALK1 (Araki et al., 2018) signaling pathways. Notably, *SGK1* has been shown to contribute to cardiac remodeling and fibrosis, and development of heart failure. These findings suggest that *SGK1* regulates blood pressure and participates in cardiovascular development and occurrence of heart failure, indicating a potential link to CHD. In this regard, we consider that *SGK1* polymorphisms may be related to the occurrence of CHD.

Furthermore, *SGK1* participates in the regulation of dendrite growth (Lang et al., 2006) and long-term memory formation (Ma et al., 2006) and contributes to the pathophysiology of several neuronal diseases including Parkinson’s disease, Alzheimer’s disease, schizophrenia, and depression (Lang et al., 2010; Miyata et al., 2015). Animal experiments have shown that the mRNA level of *SGK1* in the hippocampus of mice increased significantly under acute cold water swimming stress (Bohacek et al., 2015), suggesting that *SGK1* is closely related to stress-related mental disorders. Accumulating evidence also suggests that *SGK1* participates in the occurrence of depression *via* the glucocorticoid (Sato et al., 2008) and brain-derived neurotrophic factor (BDNF) (Lang et al., 2007) signaling pathway. Similarly, decreased hippocampal neurogenesis and structural abnormalities have been reported to occur in depressed patients owing to the up-regulation of *SGK1* (Cattaneo and Riva, 2016). *SGK1* additionally contributes to the regulation of neuroexcitability, inflammation, and oxidative stress reactions (Lang et al., 2010), which may be involved in the pathogenesis of depression. In view of the complex relationships between *SGK1*, CHD, and depression, *SGK1* is likely to be a potential co-pathogenic gene underlying susceptibility to CHD with depression.

To test this hypothesis, a case–control study was carried out to identify the role of *SGK1* variants in susceptibility to comorbidity of CHD and depression. Six candidate intron variants located in the upstream of *SGK1* gene were selected. These SNPs were reported to have a tight link with multiple disorders, including blood pressure and renin response to dietary salt intake, and type 2 diabetes (Schwab et al., 2008; Luca et al., 2009; Rao et al., 2013; Chu et al., 2015), with the possibility to affect the process of splicing, processing, and editing of mRNA. Our study of 69 CHD cases with depression and 188 cases without depression found significant differences in the genotype distribution and allele frequency of rs1743963 (A > G) and rs1763509 (G > A). For rs1743963, CHD patients with the GG genotype showed a modest but non-significant susceptibility to depression (*P* = 0.054), and the G allele was found to be a risk factor for depression in patients with CHD (*P* = 0.032). Similarly, for rs1763509, the allele A was associated with a higher risk of depression in CHD patients (*P* = 0.003). Interestingly, when patients were divided into GG and AA + AG groups according to whether they carried allele A, CHD patients in AA + AG group are more likely to have comorbidity with depression. The PHQ-9 scores in the carriers of the risk genotype for comorbid depression, AA, were significantly higher than in GG and AG carriers. In support, Chu reported that SNP rs1763509 of *SGK1* was significantly associated with blood pressure response to the intervention of dietary sodium (Chu et al., 2015). Notably, single marker association analysis is sometimes not sufficient in complex diseases, whereas haplotype-based linkage disequilibrium mapping has been considered a more informative approach for genetic association studies. In the present study, strong linkage disequilibrium was observed between the three SNPs rs1743963, rs9493857, and rs1763509 in the intron region of *SGK1* gene, and haplotype analysis suggests that the haplotype GGA is likely to be involved in the development of depression in CHD patients after Bonferroni adjustment, which may affect RNA splicing, processing, and editing. Overall, our study demonstrates for the first time the importance of *SGK1* variants in the development of depression in CHD patients. Although many genome-wide association studies (GWASs) on depression or CHD (Li et al., 2019; Liu et al., 2019; Wong et al., 2019) have been published, none of these have identified *SGK1* as a risk factor.

The remaining three SNPs, rs2758151 (Rao et al., 2013), rs9376026, and rs9389154 (Chu et al., 2015), have been reported to be associated with blood pressure response to dietary salt intake, and rs9493857 was found to regulate *SGK1* expression in response to stress (Luca et al., 2009). However, no significant differences were found between the genotypic and allelic frequencies of polymorphic sites of any of these four SNPs in our study. These negative results can be explained by the relatively small sample size, regional and racial biases, and no correction for potential population stratification, which are major limitations of the present study. Moreover, our study is also limited by the lack of a comparison group of subjects with depression but no CHD for the replication of positive results. Considering that the interactions between various genes and/or environmental factors play a part in the effects of *SGK1*, the association between *SGK1* polymorphisms and depression in CHD patients is likely to be confounded by various potential gene–gene and/or gene–environment interactions. Thus, additional association studies investigating *SGK1* diversity and susceptibility to depression in CHD patients are also required to replicate the associations. We are additionally unable to analyze the expression of *SGK1* and the functional consequence of these genetic variations. Thus, future studies are needed to further examine the effects of these SNPs on the expression of key components of *SGK1* signaling in the peripheral blood mononuclear cells of CHD patients with comorbid depression and thus confirm the relationship between *SGK1* and susceptibility to depression in CHD patients.

## Conclusion

In conclusion, the present study supports the hypothesis that *SGK1* polymorphisms contribute to the susceptibility to depression in CHD patients of the Chinese Han population. To exclude the many environmental and geographical influences on study outcomes, replication studies with large samples are needed to verify the role of these *SGK1* polymorphisms in CHD patients with comorbid depression.

## Ethics Statement

This study was carried out in accordance with the recommendations of medical ethics committee of Jining First People’s Hospital guidelines with written informed consents from all subjects. All subjects gave written informed consents and the protocol was approved by the medical ethics committee of the Jining First People’s Hospital.

## Author Contributions

PJ conceived and designed the study; HaixZ, XZ, XG, and YG were responsible for the sample collection; MY and HailZ conducted the experiments and had access to all the data in the study; WH analyzed the data and led the drafting of the manuscript; HaixZ, GL, YL, and GY provided critical revisions of the manuscript. WH and HaixZ contributed equally to the work. All authors approved the final version of the manuscript and agreed to be accountable for all aspects of the work.

## Funding

This work was supported by the National Natural Science Foundation of China (81602846, 81602724, and 81571334) and Shandong Medical and Health Science and Technology Development Program Project (2016WS0155).

## Conflict of Interest

The authors declare that the research was conducted in the absence of any commercial or financial relationships that could be construed as a potential conflict of interest.

## Supplementary Material

Supplementary Table 1 Genotyping quality assessment of the SNPs tested.

Supplementary Table 2 The information and location of SGK1 gene and these SNPs.
