# Supplementary material for: Association of SGK1 Polymorphisms With Susceptibility to Coronary Heart Disease in Chinese Han Patients With Comorbid Depression
Source: Front Genet. 2019 Oct 1;10:921. doi: 10.3389/fgene.2019.00921 (PMC6779850; doi:10.3389/fgene.2019.00921)
Supplement: Supplementary Table 1 — Genotyping quality assessment of the SNPs tested. [file Table_1.docx]

**Supplementary table 1** Genotyping quality assessment of the SNPs tested

| SNP | Genotypes | | |
| --- | --- | --- | --- |
| rs2758151 (C>T) | 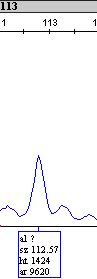  CC | 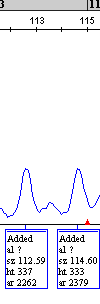  CT | 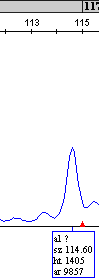  TT |
| rs1743963 (A>G) | 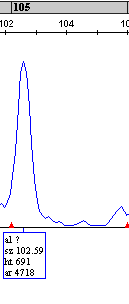  AA | 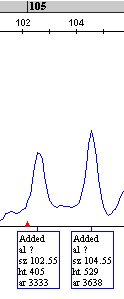  AG | 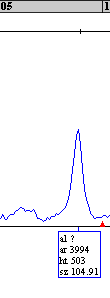  GG |
| rs9493857 (A>G) | 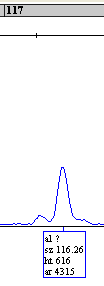  AA | 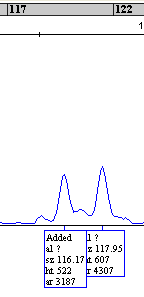  AG | 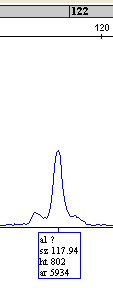  GG |
| rs1763509 (G>A) | 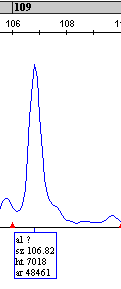  GG | 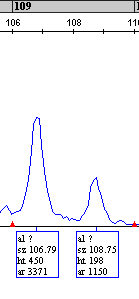  GA | 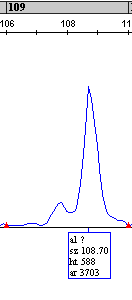  AA |
| rs9376026 (C>T) | 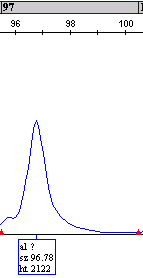  CC | 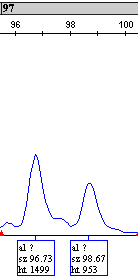  CT | 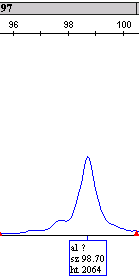  TT |
| rs9389154 (G>A) | 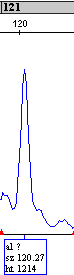  GG | 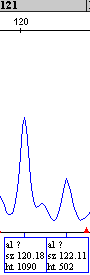  GA | 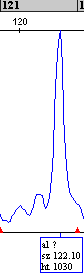  AA |
