# Supplementary material for: Association of SGK1 Polymorphisms With Susceptibility to Coronary Heart Disease in Chinese Han Patients With Comorbid Depression
Source: Front Genet. 2019 Oct 1;10:921. doi: 10.3389/fgene.2019.00921 (PMC6779850; doi:10.3389/fgene.2019.00921)
Supplement: Supplementary Table 2 — The information and location of SGK1 gene and these SNPs. [file Table_2.docx]

**Supplementary table 2** The information and location of these SNPs

| SNP | Gene | Allele | Chromosome | Position |
| --- | --- | --- | --- | --- |
| rs2758151  rs1743963  rs9493857  rs1763509  rs9376026  rs9389154 | SGK1  SGK1  SGK1  SGK1  SGK1  SGK1 | C>T  A>G  A>G  G>A  C>T  G>A | 6q23.2  6q23.2  6q23.2  6q23.2  6q23.2  6q23.2 | 134166530  134176537  134209559  134233200  134281316  134293623 |
